# Microscopic revelation of the solid–gas coupling and Knudsen effect on the thermal conductivity of silica aerogel with inter-connected pores

**DOI:** 10.1038/s41598-022-24133-5

**Published:** 2022-12-05

**Authors:** Jing Liu, Piyapong Buahom, Chang Lu, Haiyan Yu, Chul B. Park

**Affiliations:** 1 College of Vehicle and Traffic Engineering, Henan University of Science and Technology, Luoyang, 471003 China; 2 Department of Mechanical and Industrial Engineering, University of Toronto, 5 King’s College Road, Toronto, ON M5S 3G8 Canada; 3 Department of Mechanical Engineering, McMaster University, 1280 Main Street West, Hamilton, ON L8S 4L8 Canada; 4 School of Energy Science and Engineering, Harbin Institute of Technology, Harbin, 150001 China

**Keywords:** Structural properties, Statistical physics, thermodynamics and nonlinear dynamics

## Abstract

As a star insulation material, aerogel plays a significant role in saving energy and meeting temperature requirements in industry due to its extremely low thermal conductivity. The prediction of aerogel’s thermal conductivity is of great interest in both research and industry, particularly because of the difficulty in measuring the separated gas conductivities directly by experiment. Hence, the proportions of separated gas conduction and solid–gas coupling conduction are debatable. In this work, molecular dynamics simulations were performed on porous silica aerogel systems to determine their thermal conductivities directly. The pore size achieved in the present study was improved significantly, making it possible to include the gas phase in the investigation of aerogel thermal conductivity. The separated solid conductivity ${\lambda }_{s}$ and the separated gas thermal conductivity ${\lambda }_{g}$ as well as the effective solid conductivity ${\lambda }_{s}^{e}$ and the effective gas conductivity ${\lambda }_{g}^{e}$ were calculated. The results suggest that the solid–gas coupling effect is negligible in rarefied gas because the enhancement of thermal conduction due to the short cut bridging effect by gas between gaps in the solid is limited. The gas pressure is the most significant factor that affects the solid–gas coupling effect. The large differential between the prediction and the actual value of the thermal conductivity is mainly from the underestimate of ${\lambda }_{g}$, and not because of ignoring the coupling effect. As a conclusion, the solid–gas coupling effect can be neglected in the prediction of silica aerogel’s thermal conductivity at low and moderate gas pressure, i.e., decreasing the gas pressure is the most efficient way to suppress the coupling effect. The findings could be used in multi-scale simulations and be beneficial for improving the accuracy of predictions of aerogel thermal conductivity.

## Introduction

Aerogel is a synthetic material comprised of a nanoporous solid and a dispersed gas phase. It is derived from a gel, where the liquid component will be extracted and replaced by gas^1^. The result is a structure composed of a gel-like solid network that contains air pockets of various sizes. The air pockets often take up the majority of space within the material, which leads to several special properties such as light weight, low thermal conductivity and high specific surface area. These excellent properties make aerogel a star material in a broad area of industry, including building, energy, catalysis, aeronautics and astronautics. One of the most desirable properties that aerogel possesses is the extremely low thermal conductivity. As a new type of insulation material, aerogel plays a significant role in saving energy and meeting temperature requirements. In NASA’s space exploration programs, Mars Rovers used aerogel as thermal insulation which was considered as one of the key factors to the success of the program^2^. In civilian fields, aerogel also offers great application potential and the market share of aerogel products is growing rapidly^3^. As the application need of aerogel increases, the mechanisms underlying its incredible thermal insulation performance are attracting more and more interest from researchers.

Silica aerogel is the most common type of aerogel. The density of silica aerogel is as low as 0.001 ${\text{g}}\,{\text{cm}}^{ - 3}$, and the lowest thermal conductivity it can reach is below 0.02 $\mathrm{W }{\mathrm{m}}^{-1} {\mathrm{K}}^{-1}$ in air and about 0.004 $\mathrm{W }{\mathrm{m}}^{-1} {\mathrm{K}}^{-1}$ in vacuum at room temperature. Heat transfer in aerogels is dominated by conduction and radiation while convection is largely prevented because gas cannot circulate well through the nanoporous structure^4^. Recently, Fong^5^ considered quantum fluctuation as the fourth way of heat transfer but it is negligible in aerogels. Silica aerogel has especially good thermal insulating performance because gas and silica, which it is composed of, are both poor thermal conductors. And the radiation is effectively attenuated but not negligible compared to the heat conduction when it comes to the nanoporous materials^6–8^ like aerogel. When studying the thermal conductivity of aerogel, heat conduction and radiation are the only two basic heat transfer modes that are involved.

The total thermal conductivity of aerogel ${\lambda }_{total}$ is usually considered as the superposition of contributions from heat conduction through the solid and the gas and thermal radiation according to the widely used formula^9^:

1 $${\lambda }_{total}={\lambda }_{s}+{\lambda }_{g}+{\lambda }_{r},$$

where ${\lambda }_{s}$ and ${\lambda }_{g}$ are the solid conductivity and the gas conductivity, respectively, and ${\lambda }_{r}$ is the radiative conductivity. The direct superposition formula has a simple form and is convenient to calculate, so it is applied by many scholars^10–12^. The gas conductivity ${\lambda }_{g}$ accounts for up to 62% of the total thermal conductivity ${\lambda }_{total}$^13^. The value of ${\lambda }_{g}$ cannot be measured directly by experiment and is usually obtained by subtracting the total conductivity in vacuum ${\lambda }_{vac}$ from the total conductivity measured at a certain gas pressure ${\lambda }_{total}$. It is worth noting that the sum of the solid conductivity ${\lambda }_{s}$ and the radiative conductivity ${\lambda }_{r}$ can be obtained as the total conductivity in vacuum ${\lambda }_{vac}$. In the prediction of the gas conductivity ${\lambda }_{g}$, Kaganer’s^14^ model is often used because it takes the Knudsen effect in nano pores into consideration through the Knudsen number *Kn*:

2 $$\lambda_{g} = \frac{{\lambda_{g}^{0} }}{{1 + C_{g} \cdot Kn}},$$

where ${\lambda }_{g}^{0}$ is the conductivity of gas in free space and *Kn* is the ratio of the mean free path of gas molecules ${l}_{mean}$ to the characteristic length ${l}_{c}$. ${l}_{mean}$ is determined by the gas pressure and ${l}_{c}$ is usually set as the pore size $D$ for a nanoporous structure:$Kn=\frac{{l}_{mean}}{D}$. ${C}_{g}$ is a constant associated with the gas species^15^ given by:

3 $$C_{g} = 2 \cdot \frac{2\gamma }{{\gamma + 1}} \cdot \frac{2 - \alpha }{\alpha } \cdot \frac{1}{Pr},$$

where $\gamma$ is the specific heat ratio, $\alpha$ is the thermal accommodation coefficient and $Pr$ is the Prandtl number. Based on the gas kinetics theory for gas molecules between two parallel plates, the value of ${C}_{g}$ obtained from Eqs. (2) and (3) is around 3.2 for air and 3.6 for argon^16^.

Notably, the experimental results are fitted through adjusting ${C}_{g}$ based on Eq. (2)^17,18^. Recent work by Li^19^ obtained a fitted value of ${C}_{g}=2.0$ for argon in aerogel based on the fitting results from the Direct Simulation Monte Carlo (DSMC) method. As mentioned above, using the ideal ${C}_{g}$ by Eq. (3) and the Knudsen formula Eq. (2), the predicted effective thermal conductivity by Eq. (1) is often found to be underestimated when compared with the experimental data^16^. According to some theories^16,20,21^ introduced to explained this observation, the main reason for such underestimation could be that the contribution of coupling heat transfer between solid and gas is ignored. Swimm^16^ claims that heat does not transfer through solid and gas phases completely in a parallel manner in aerogel due to the tortuous and complex structure. At sites of point contact and dangling bonds (particulate necks), some gas molecules are trapped, and the heat transfer path can be considered as serial through the solid–gas-solid path. Bi et al.^20^ suggested that the prediction of gas conductivity without considering this solid–gas coupling effect could result in an underestimate of around 30% for the measured thermal conductivity for silica aerogel with a density of 0.12 g/cm^3^. On the other hand, in a reverse problem to determine the pore size based on the Knudsen formula Eq. (2) by considering the dependency of the gas thermal conductivity on the gas pressure, the mean pore size *D* obtained will be 10 times larger than the actual value if the coupling effect is ignored^22^. Bi^20^ and Zhao^21^ considered that the quasi-lattice vibrating gas molecules in the immediate proximity of the contact point of adjacent secondary solid particles act as thermal short cut bridges and contribute significantly to the solid–gas coupling effect. However, it is still a matter of debate as to how much influence this coupling effect has on the effective conductivity of aerogel. Several studies^16,20,21^ exist that provide insights into the solid–gas coupling effect in aerogel. However, various assumptions including simplification of porous structure and thermal transport phenomena are required. Also, the crucial details of thermal transport at the atomic level, where the energy exchanges through the interaction between solid and gas atoms, are excluded.

To take such detailed phenomena at the atomic level into account, molecular dynamics (MD) simulation, which is based on the classical mechanics with limited assumptions, has been used to reveal some microscopic aspects of the solid–gas coupling heat transfer in aerogel. As a fast-developing method, MD simulation also plays an important role in improving the prediction of thermal conductivity and could be applied alone or as a part of multi-scale and integration simulation. It should be noted that the thermal radiation is not easily considered in MD simulation unless the first principles molecular dynamics method^23^ is used, due to the totally different mechanism of heat transfer. Fortunately, the radiative conduction is strongly related to the temperature^24^, resulting in a low radiative conductivity at room temperature or below^25^, which covers many use scenarios. Furthermore, several approaches are used to reduce the radiative heat transfer such as loading fibers or opacifiers into aerogels^26,27^. All these conditions where the radiative conductivity is negligible compared to the total thermal conductivity can be simulated by classical MD simulation. MD simulation is a very promising method and has attracted more and more interest for its convenience in discovering the mechanisms in thermal and mechanical properties of nanomaterials^28,29^. The latest developments in computer technology make it affordable to simulate the thermal conduction of nanomaterials on the scale of tens to hundreds of nanometers.

At present, the application of MD simulation to investigate thermal transport in aerogels is focused on conduction through the solid. Coquil^30^ applied MD to the prediction of the thermal conductivity of amorphous nanoporous silica for the first time. They introduced pores in amorphous silica matrix by removing atoms within selected regions and investigated the influences of pore diameter and porosity on the solid thermal conductivity. Their results suggested that the thermal conductivity of amorphous nanoporous silica was independent of the pore diameter and depended only on porosity. It should be noted that the spherical pores were monodisperse and organized in a simple cubic lattice in Coquil’s study, which differs greatly from the more realistic fractal geometry of silica aerogel material. Soon afterwards, Ng et al.^31^ modeled relatively reasonable silica aerogel structures in an MD simulation and determined their thermal conductivity. Porous aerogels with density between 300 and 1 g cm^−3^ were rendered through negative pressure rupturing (NPR) of dense amorphous silica. Non-equilibrium MD simulations were performed and the well-known BKS (van Beest, Kramer and van Santen) potential was used to describe the interaction between silicon and oxygen in silica. The thermal conductivity they obtained was of the same order of magnitude as that of bulk sintered aerogel and varied almost linearly with density. However, it was found by experiment^28,29^ that the variation of solid thermal conductivity of aerogel can fit a power law function: ${\lambda }_{s}=Const\cdot {\rho }^{b}$, where *b* is in the range of 1.5–1.6^32,33^. Ng et al.^34^ switched the interaction potential to the Tersoff potential in their later work and successfully obtained a closer fit of the power law relation between thermal conductivity and density, where the value of *b* is 1.61. Liu et al.^35^ modeled a smaller basic heat transport unit of aerogel solid skeleton, which consists of two adjacent secondary particles and studied the thermal resistance of these two contact particles. Their results indicated that the heat conduction in the solid is constrained by small contact length between particles as well as the defects in silica. These researches demonstrate that the prediction of aerogel’s thermal properties through MD simulation method is possible. However, it is worth emphasizing that the MD simulation studies in this field are still scarce and only focused on the conduction in the solid phase. Due to the small achievable pore size that can only contain a minimal number of gas molecules, gas conduction was barely included in previous MD studies. This is clearly inconsistent with the common scenario in industrial applications. To draw a full picture of the heat conduction through the solid and the gas in aerogels and to further improve the accuracy of the model by including the solid–gas coupling effect, MD simulations including both solid and gas phase are essential.

In the present study, different species of gas were introduced into silica aerogel and MD simulations were performed to reveal the details of the separated and coupling solid and gas thermal conductivity. The significance of the solid–gas coupling effect, which is indirect and unclear in the macroscopic experiments, was evaluated by numerical experiment. The influence of the factors such as aerogel density $\rho$(and its corresponding pore size), the defect concentration ${C}_{def}$(imperfections in the silica network), the species of gas and the gas pressure on the solid–gas coupling effect were measured to provide a better informed view of the thermal conduction of aerogel.

## Methods

All the simulations were performed using classical molecular dynamics code Large-scale-Atomic/Molecular Massively Parallel Simulator (LAMMPS)^36^. There are three critical steps in the simulations which are the determination of the interaction potentials, the simulation box preparation and the final measurement runs. The potentials that describe the interactions of the atoms in the simulation box were determined first. Then, the simulation box was prepared. Each box consisted of solid and gas phases. The material of the solid phase was silica, and the gas phase was chosen as helium (He), methane (CH_4_), argon (Ar), nitrogen (N_2_) and carbon dioxide (CO_2_). The initial simulation box should go through a series of preparation runs to reach the desired porous structure with different densities, after which the reverse non-equilibrium molecular dynamics (RNEMD) simulations were conducted on these aerogel systems to determine the thermal conductivity. Details are introduced as follows: interaction potential (“Interaction potential: BKS+24-6 L-J, Tersoff and 12-6 L-J potential”), simulation box preparation (“Simulation box preparation”) and the RNEMD procedure (“Reverse non-equilibrium molecular dynamics (RNEMD) procedure”).

### Interaction potential: BKS + 24–6 L–J, Tersoff and 12–6 L–J potential

Two interaction potentials were used in tandem for silica. When generating the porous silica aerogel structure, the modified two-body potential Beest, Kramer and van Santen (BKS)^37^ potential for the solid phase was applied. The conventional BKS potential has good performance for maintaining the cohesion of the system. To prevent the atoms approaching each other too closely, a 24–6 Lennard–Jones (24–6 L–J) potential^31^ was added to the BKS potential as follows:

4 $$V\left( {r_{ij} } \right) = \frac{{q_{i} q_{j} }}{{r_{ij} }} + A_{ij} \exp \left( { - \frac{{r_{ij} }}{{B_{ij} }}} \right) - \frac{{C_{ij} }}{{r_{ij}^{6} }} + 4\varepsilon_{ij} \left[ {\left( {\frac{{\sigma_{ij} }}{{r_{ij} }}} \right)^{24} - \left( {\frac{{\sigma_{ij} }}{{r_{ij} }}} \right)^{6} } \right].$$

In the NEMD run, the re-parameterized three-body potential, Tersoff^38,39^, was applied to improve the accuracy as follows:

5 $$E = \sum\limits_{i} {E_{i} } = \frac{1}{2}\sum\limits_{i \ne j} {V_{ij} } ,$$

where

6 $$V_{ij} \left( {r_{ij} } \right) = f_{c} \left( {r_{ij} } \right)\left[ {f_{R} \left( {r_{ij} } \right) + b_{ij} f_{A} \left( {r_{ij} } \right)} \right],$$

7 $$f_{R} \left( {r_{ij} } \right){ = }P_{ij} \exp \left( { - \gamma_{ij} r_{ij} } \right),f_{A} \left( {r_{ij} } \right) = Q_{ij} \left( { - \mu_{ij} r_{ij} } \right),$$

8 $$f_{c} \left( {r_{ij} } \right) = \left\{ {\begin{array}{*{20}l} {1,} \hfill & {r_{ij} < R_{ij} } \hfill \\ {\frac{1}{2} + \frac{1}{2}\cos \left( {\pi \frac{{r_{ij} - R_{ij} }}{{S_{ij} - R_{ij} }}} \right),} \hfill & {R_{ij} < r_{ij} < S_{ij} } \hfill \\ {0,} \hfill & {r_{ij} > S_{ij} } \hfill \\ \end{array} } \right.,$$

9 $$b_{ij} = \chi_{ij} \left( {1 + \beta_{i}^{{n_{i} }} \zeta_{ij}^{{n_{i} }} } \right)^{{ - \frac{1}{{2n_{i} }}}} ,$$

10 $$\zeta_{ij} = \sum\limits_{k \ne i,j} {f_{c} \left( {r_{ik} } \right)} \omega_{ik} g\left( {\theta_{ijk} } \right),g\left( {\theta_{ijk} } \right) = 1 + \frac{{c_{i}^{2} }}{{d_{i}^{2} }} - \frac{{c_{i}^{2} }}{{d_{i}^{2} + \left( {h_{i} - \cos \theta_{ijk} } \right)^{2} }},$$

11 $$\gamma_{ij} = \frac{{\gamma_{i} + \gamma_{j} }}{2},\mu_{ij} = \frac{{\mu_{i} + \mu_{j} }}{2},$$

12 $$P_{ij} = \left( {P_{i} + P_{j} } \right)^{\frac{1}{2}} ,Q_{ij} = \left( {Q_{i} + Q_{j} } \right)^{\frac{1}{2}} ,$$

13 $$R_{ij} = \left( {R_{i} R_{j} } \right)^{\frac{1}{2}} ,S_{ij} = \left( {S_{i} S_{j} } \right)^{\frac{1}{2}} .$$

The re-parameterized Tersoff potential can reproduce the thermal conductivity of bulk amorphous silica very closely to the experimental data based on the present simulation results. Hence, modelling the thermal characteristics of silica using this method gives more accurate results than using the combination of BKS and 24–6 L–J potentials, reducing the overestimation from 55%^34^ to less than 4%. The BKS and Tersoff potential parameters for the solid phase are summarized in Tables 1 and 2, respectively.

**Table 1:** Parameters in the BKS + 24-6L-J potential for the solid phase.

Parameter	Si–Si	Si–O	O–O
$A$(eV)	0.0	18,003.7572	1388.7730
$B$(Å)	0.1	0.205205	0.362319
$C$(${\text{eV}}\,{\text{\AA}}^{6}$)	0.0	133.5381	175.0
$\varepsilon$(eV)	13.20	$1.12 \times 10^{ - 2}$	$4.78 \times 10^{ - 4}$
$\sigma$(Å)	0.40	1.35	2.20
$q$	$q_{Si} = 2.4{\text{e}}$, $q_{O} = - 1.2{\text{e}}$		

Parameters in the BKS + 24–6L–J potential for the solid phase are from Ref.^31^.

**Table 2:** Parameters in the Tersoff potential for the solid phase.

Parameter	Si	O
$P$ (eV)	$1.8308 \times 10^{3}$	$1.88255 \times 10^{3}$
$Q$ (eV)	$4.7118 \times 10^{2}$	$2.18787 \times 10^{2}$
$\gamma$ (${\text{\AA}}^{ - 1}$)	2.4799	4.17108
$\mu$ (${\text{\AA}}^{ - 1}$)	1.7322	2.35692
$\beta$	$1.1000 \times 10^{ - 6}$	$1.1632 \times 10^{ - 7}$
$n$	$7.8734 \times 10^{ - 1}$	$1.04968$
$c$	$1.0039 \times 10^{5}$	$6.46921 \times 10^{4}$
$d$	$1.6217 \times 10^{1}$	$4.11127$
$h$	$- 5.9825 \times 10^{ - 1}$	$- 8.45922 \times 10^{ - 1}$
$R$ (${\text{\AA}}$)	2.5	1.7
$S$ (${\text{\AA}}$)	2.8	2.0
$\chi_{Si - O}$	1.17945	

Parameters in the Tersoff potential for the solid phase are from^38,39^.

For the gas phase, 12–6 Lennard–Jones (12–6 L–J) potential was used to describe the interaction between gas molecules and between gas and silica.

14 $$V\left( {r_{ij} } \right) = 4\varepsilon_{ij} \left[ {\left( {\frac{{\sigma_{ij} }}{{r_{ij} }}} \right)^{12} - \left( {\frac{{\sigma_{ij} }}{{r_{ij} }}} \right)^{6} } \right],$$

where the 12–6 L–J parameters for different gas species are adopted from Ref.^40^, as shown in Table 3.

**Table 3:** Parameters in the 12–6 L–J potential for the gas phase.

Gas type	$\varepsilon_{ii}$ (eV)	$\sigma_{ii}$ (Å)	$\varepsilon_{i - O}$ (eV)	$\sigma_{i - O}$ (Å)	$\varepsilon_{i - Si}$ (eV)
He	$9.38 \times 10^{ - 4}$	2.640	$2.411 \times 10^{ - 3}$	2.952	0
${\text{CH}}_{{4}}$	$1.303 \times 10^{ - 2}$	3.737	$8.997 \times 10^{ - 3}$	3.501	
${\text{N}}_{{2}}$	$8.626 \times 10^{ - 3}$	3.613	$7.326 \times 10^{ - 3}$	3.439	
Ar	$1.031 \times 10^{ - 2}$	3.405	$8.007 \times 10^{ - 3}$	3.335	
${\text{CO}}_{{2}}$	$2.081 \times 10^{ - 2}$	3.673	$1.137 \times 10^{ - 2}$	3.469	

The12–6 L–J parameters for different gas species are adopted from Ref.^40^.

### Simulation box preparation

**Figure 1:**
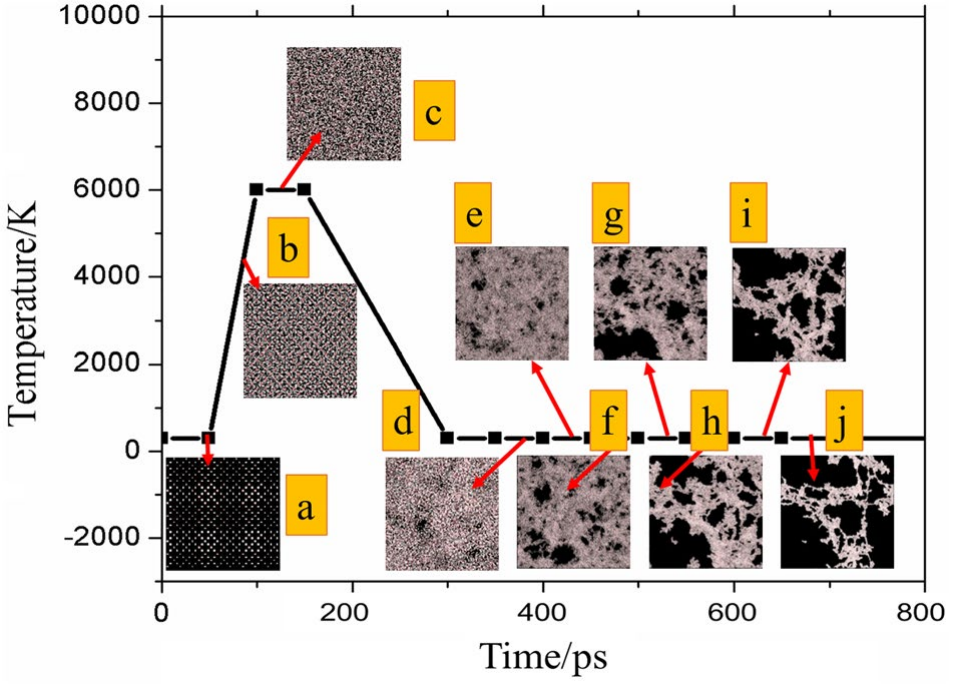
Snapshots of the simulation box at every stage to construct the porous aerogel: (**a**) crystalline *β*-cristobalite, (**b**) melting silica, (**c**) amorphous dense silica, and (**d**–**j**) porous silica.

The porous silica aerogel structures were generated using the negative pressure rupture (NPR) method^41^ through the following steps. First, the ideal configuration of crystalline *β*-cristobalite containing 192,000 atoms (64,000 silicon and 128,000 oxygen atoms) was prepared. The lattice constant of *β*-cristobalite is 7.16 Å and the corresponding density is 2.170 ${\text{g}}\,{\text{cm}}^{ - 3}$^42^. The crystal was melted by heating to 6000 K in 50 ps and held at that temperature for another 50 ps to relax the system. Then, it was quenched to 300 K to obtain the amorphous silica. The pressure was kept constant during the whole annealing process. Subsequently, the dense silica was expanded by 1.2 times along each direction so as to reach the target volume successively. This is the so-called Negative Pressure Rupture (NPR) method since this expansion leads to the rupture of Si–O bonds under negative pressure^41^. Each step of expansion was followed by an equilibration run of 50 ps at 300 K so as to obtain the porous silica skeleton structures. The final density was in the range of 0.0557–0.482 ${\text{g}}\,{\text{cm}}^{ - 3}$. The corresponding porosity is between 0.318 and 0.974. Figure 1 clearly displays the temperature maintained during the constructing process. Defect concentration $C_{def}$ reflects imperfections in the solid structure affecting the solid conductivity. Different concentrations of defects $C_{def}$ were introduced by deleting atoms randomly, as in Liu’s work^35^. The maximum defect concentration in the present study was 0.30. The simulation box length and the composition of each skeleton systems are shown in Table 4.

**Table 4:** The simulation box length and compositions of the skeletons *d*–*j* with different defect concentration $C_{def}$.

Defect concentration	Composition^a^		Skeleton						
			(d)	(e)	(f)	(g)	(h)	(i)	(j)
			Box length(Å)						
			162.67	195.21	234.25	281.10	337.32	404.78	485.74
			Density $\rho$ (${\text{g}}\,{\text{cm}}^{ - 3}$)						
	$N_{Si}$	$N_{O}$							
$C_{def}$ = 0.00	64,000	128,000	1.4803	0.85666	0.49575	0.28689	0.16603	0.09608	0.0556
$C_{def}$ = 0.10	≈ 57,600	≈ 11,520	1.33227	0.77099	0.44617	0.2582	0.14942	0.08647	0.05004
$C_{def}$ = 0.20	≈ 51,200	≈ 10,240	1.18424	0.68533	0.3966	0.22951	0.13282	0.07686	0.04448
$C_{def}$ = 0.30	≈ 44,800	≈ 89,600	1.03621	0.59966	0.34702	0.20082	0.11622	0.06726	0.03892

^a^$N_{Si}$: the number of silicon atoms;$N_{O}$: the number of oxygen atoms.

**Figure 2:**
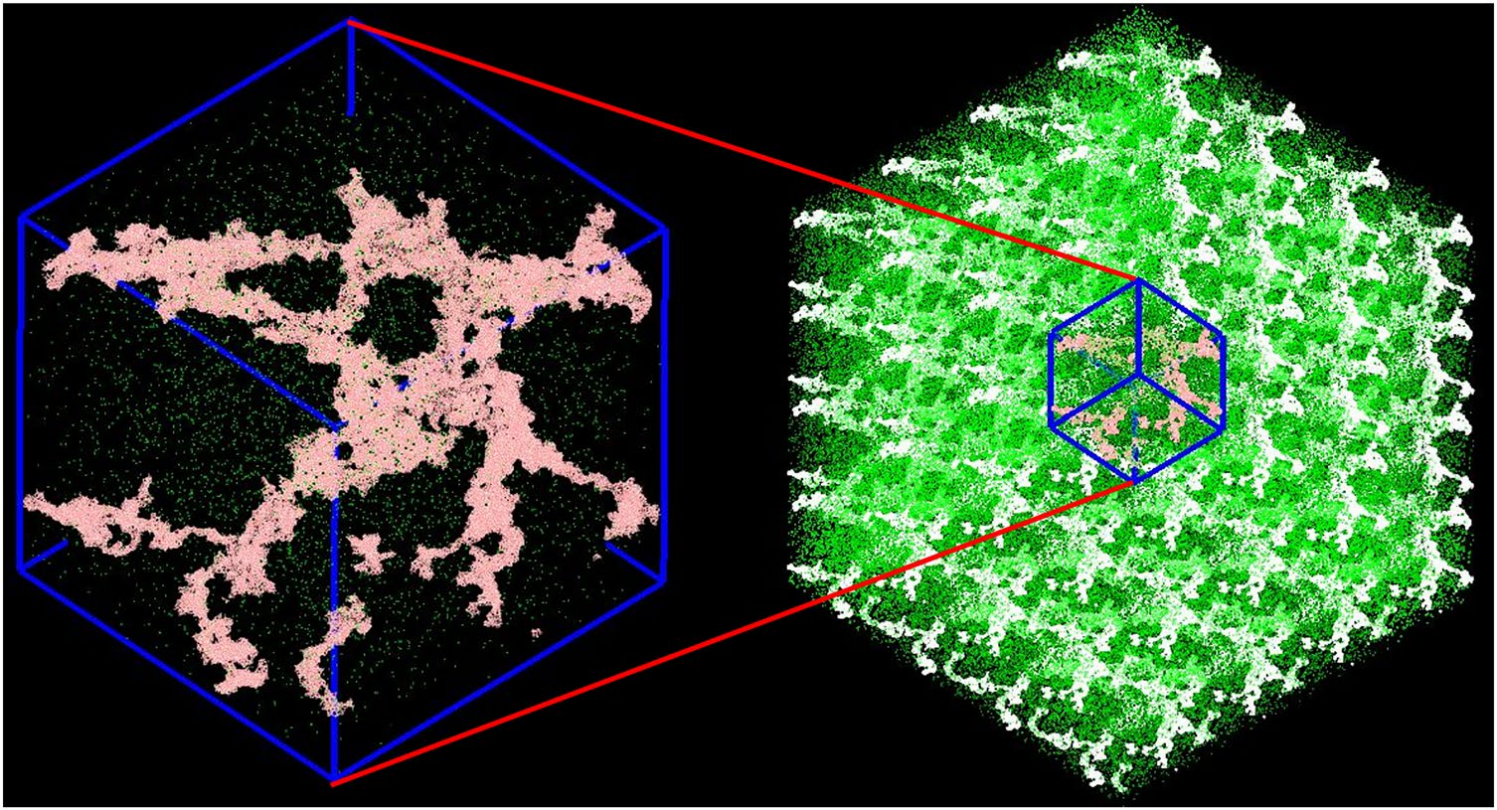
Schematic drawing of the periodic boundary conditions. The simulation box is shown on the left. The large system (right) consisting of an infinite number of repeating unit cells (left) represents the macro-scale structure of the aerogel.

During the generating process, the modified BKS (BKS + 24–6 L–J) potential was used. The cutoff of short-range interaction was set at 10 Å, beyond which the long-range Coulombic interactions were calculated in reciprocal space via LAMMPS' particle–particle particle-mesh solver (PPPM)^43^. The accuracy, that is, the relative root-mean-square error in per-atom forces was specified as $10^{ - 5}$. Then, the BKS potential was removed, and re-parameterized Tersoff potential was applied. The silica skeleton structures were relaxed through heating up to 3000 K in 50 ps and equilibration at 3000 K for another 50 ps. Then, the temperature dropped to 300 K, followed by an equilibration run of 50 ps. The porous structures obtained were used to determine the solid thermal conductivity through non-equilibrium molecular dynamics, after which the void spaces were filled with different species of gas and the total thermal conductivity was measured. The cutoff was set to 8 Å for L–J interactions between silica and gas and 35 Å for L–J between gas and gas. The time step was 0.5 fs. Periodic boundary conditions^44^ were applied in three dimensions, as shown in Fig. 2, to overcome the surface effect during all the simulations.

### Reverse non-equilibrium molecular dynamics (RNEMD) procedure

**Figure 3:**
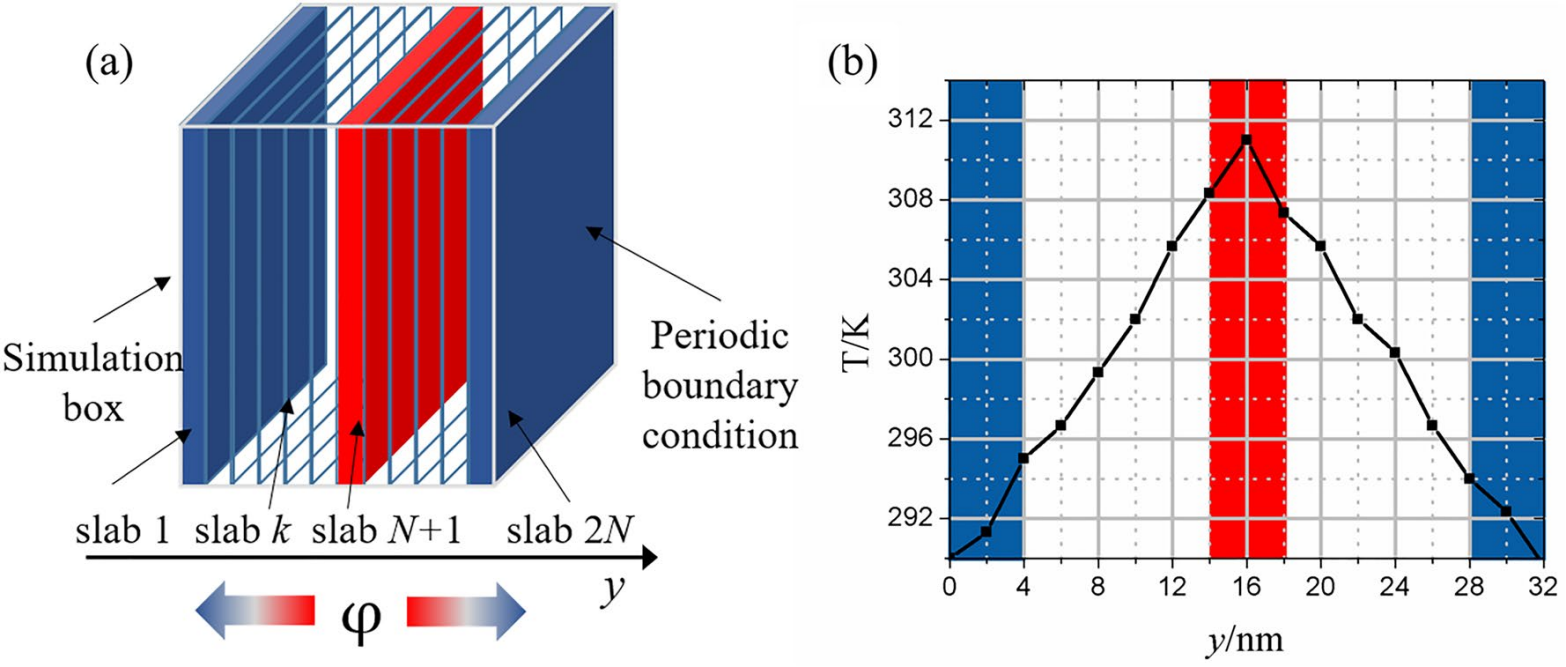
(**a**) A schematic drawing of reverse non-equilibrium MD, and (**b**) the temperature gradient profile along the heat transfer direction.

According to Fourier’s Law, $\lambda { = } - \frac{\varphi }{\partial T/\partial y}$, the total thermal conductivity $\lambda$ of an aerogel is dependent on the temperature gradient in the direction of heat transfer $\partial T/\partial z$ and the heat flux $\varphi$. To calculate the thermal conductivity, a reverse non-equilibrium molecular dynamics simulation (RNEMD) approach, which is the Muller–Plathe algorithm^45^, was adopted by dividing the whole simulation box into 2* N* slabs with identical thicknesses along the transversal direction (*y* axis), as shown in Fig. 3. Then, the instantaneous local kinetic (absolute) temperature $T_{k}$ in slab *k* was determined by the average of the molecules’ kinetic energy, as follows:

15 $$T_{k} = \frac{1}{{3n_{k} k_{B} }}\sum\limits_{i \in k}^{{n_{k} }} {m_{i} v_{i}^{2} } ,$$

where $n_{k}$ is the number of atoms in slab *k*, $m_{i}$ and $v_{i}$ are the mass and velocity of atom *i* in slab *k,* respectively, and $k_{B}$ is Boltzmann’s constant. Hence, the temperature gradient can be represented as $\partial T/\partial y{ = }T_{N + 1} - T_{1}$. Considering that the flux of energy is imposed through exchanging kinetic energy between the hottest particle in slab 1 and the coldest particle in slab *N* + 1 at regular intervals, the average heat flux $\varphi$ can be written as follows:

16 $$\varphi { = }\frac{1}{{2tL_{x} L_{z} }}\sum\limits_{{N_{swap} }} {\frac{{\left( {m_{hot} v_{hot}^{2} - m_{cold} v_{cold}^{2} } \right)}}{2}} ,$$

where *t* is the time over which the heat flux is imposed.$L_{x}$ and $L_{z}$ are, respectively, the lengths along *x* and *z* axes of the simulation box, and $N_{swap}$ is the number of energy swaps. Finally, a relatively steady temperature gradient was induced as a response. A schematic drawing of NREMD is shown in Fig. 3.

## Results and discussion

### Validation of the force field

**Figure 4:**
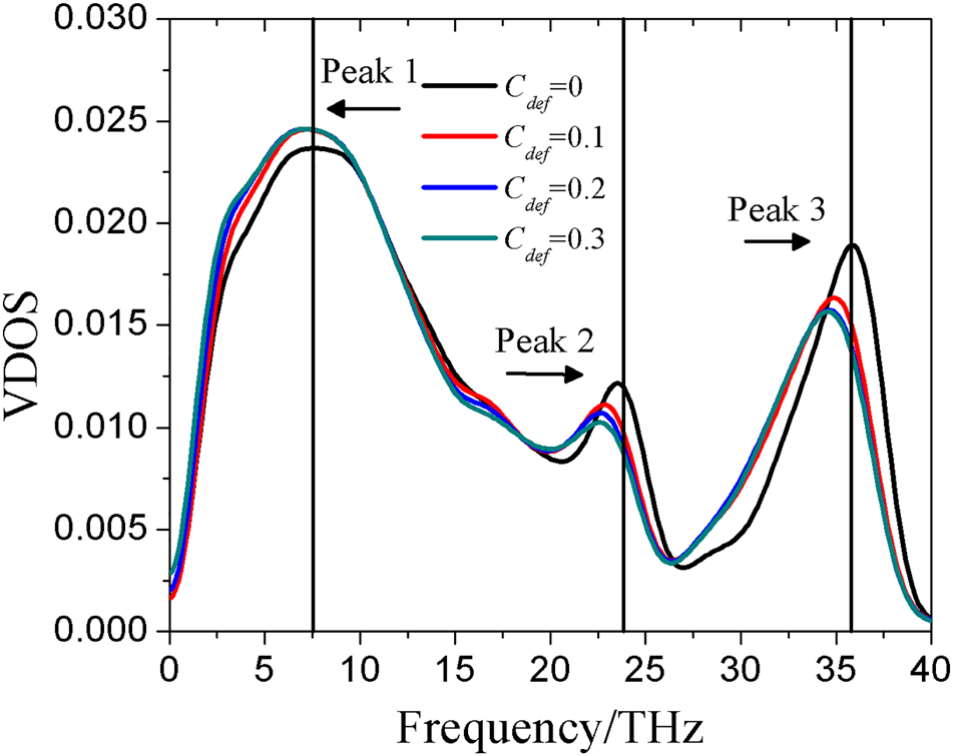
VDOS of the bulk amorphous silica with different defect concentration $C_{def}$.

To validate the reliability of the re-parameterized Tersoff potential, the vibrational density of states (VDOS) of bulk amorphous silica was first extracted from the simulation results and examined. VDOS was calculated based on the Fourier transform of the velocity autocorrelation function (VACF). VDOS of a solid is a reflection of vibrational modes of atoms, which is determinant to the thermal conduction behavior. Figure 4 shows the vibrational spectrum of the bulk silica. For VDOS of amorphous silica, three significant peaks are observed, located near 7.5 THz, 24 THz and 36 THz. Except the location of the first peak, which is perfectly consistent with simulation^34^ but a little left-shift compared with the experimental value 10 THz^46^, the VDOS observed in the present study is in good agreement with the results from both the previous experiment^46^ and simulation^34^. The first two peaks are associated with the transverse acoustic (TA) and longitudinal acoustic (LA) branches, respectively. The third peak at 36 THz corresponds to the high frequency vibrational modes, the contribution of which can be ignored due to their low group velocities^35^. Therefore, the TA and LA modes contribute most to the heat conduction of the solid. Moreover, the thermal conductivity of bulk amorphous silica where the solid conductivity accounts for 100% is obtained as $$1.44 \pm 0.04$$$${\text{W}}\,{\text{m}}^{{{ - }1}} \,{\text{K}}^{{{ - }1}}$$, consistent with the experimental data which is in the range of 1.37–1.41 $${\text{W}}\,{\text{m}}^{{{ - }1}} \,{\text{K}}^{{{ - }1}}$$ ^47^. This indicates that the Tersoff potential is applicable to the thermal characterization of silica.

The effect of defect concentration $C_{def}$ on the solid thermal conductivity of bulk amorphous silica is also shown in Fig. 4. At higher defect concentration, all three peaks shift to lower frequency, while the heights of TA modes increase and LA modes decrease. As calculated in the present study, the solid thermal conductivities of bulk amorphous silica with $C_{def}$ = 0, 0.10, 0.20 and 0.30 are 1.44 ${\text{W}}\,{\text{m}}^{{{ - }1}} \,{\text{K}}^{{{ - }1}}$, 1.21 ${\text{W}}\,{\text{m}}^{{{ - }1}} \,{\text{K}}^{{{ - }1}}$, 0.966 ${\text{W}}\,{\text{m}}^{{{ - }1}} \,{\text{K}}^{{{ - }1}}$ and 0.870 ${\text{W}}\,{\text{m}}^{{{ - }1}} \,{\text{K}}^{{{ - }1}}$, respectively. Given that the thermal conductivity reduces with rising defect concentration, it can be derived that the constraint of LA modes is the reason for lower thermal conductivity of a defective silica skeleton.

### Solid thermal conductivity ${\lambda }_{s}$

**Figure 5:**
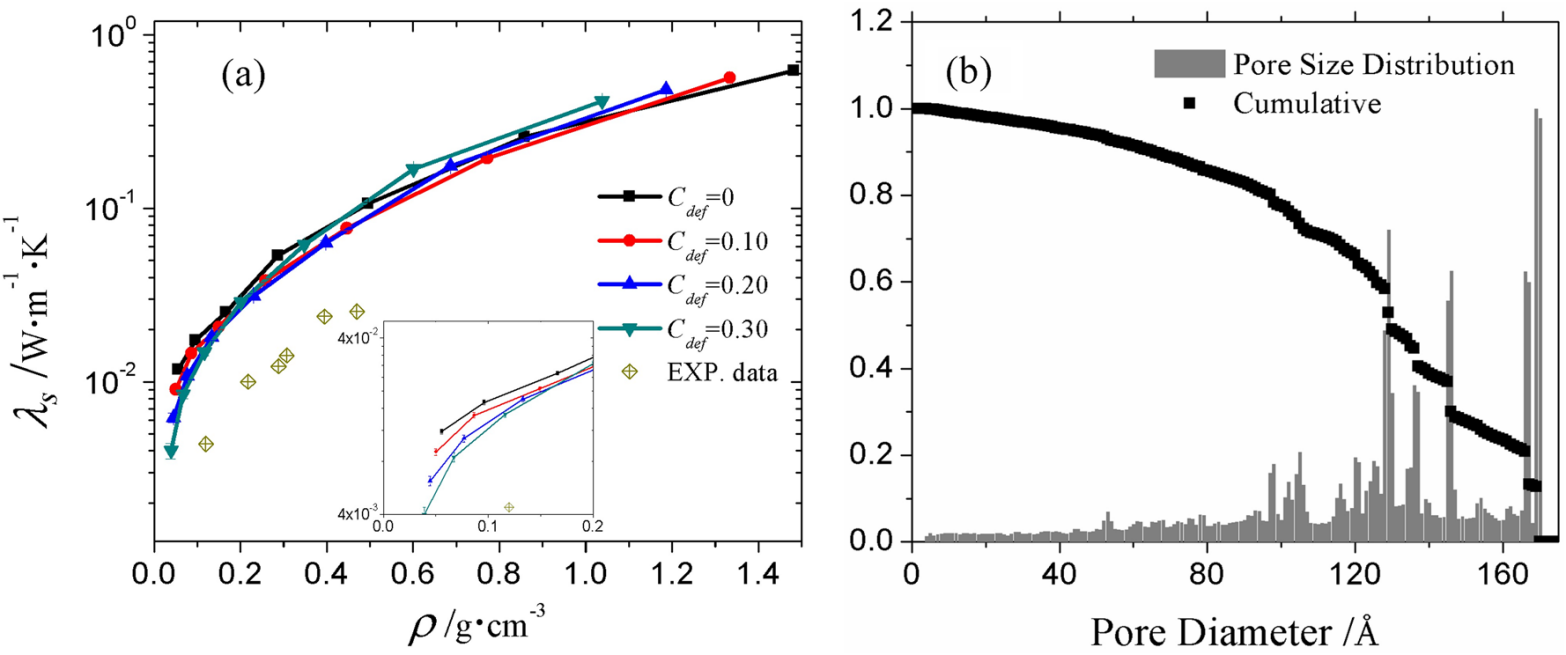
(**a**) The solid thermal conductivity ${\lambda }_{s}$ as a function of aerogel density $\rho$ at various defect concentrations together with experimental data from reference^20^, (**b**) The pore size distribution of the aerogel solid skeleton at $\rho$ = 0.0557 ${\text{g}}\,{\text{cm}}^{ - 3}$ and $C_{def}$ = 0.0.

The solid thermal conductivities ${\lambda }_{s}$ were calculated without any gas molecules filled. Here, ${\lambda }_{s}$ is the effective thermal conductivity of aerogel in absolute vacuum without consideration of radiative conductivity. By virtue of MD and NPR methods, the fractal nature of silica aerogel structure can be reproduced. Based on percolation theory^48^, ${\lambda }_{s}$ is related to the density and fractality of the aerogel. The ${\lambda }_{s}$ obtained, as shown in Fig. 5a, can be fitted to a power law function of density as follows:

17 $$\lambda_{s} = \alpha \rho^{b} ,$$

where the factor *b* is influenced by the fractal dimension of the aerogel structure, which is decided by the processing conditions. For solid skeletons with defect concentration $C_{def}$ of 0, 0.10, 0.20 and 0.30, ${\lambda }_{s}$ systematically increases with increasing density and is proportional to ${\rho }^{1.57}$, ${\rho }^{1.58}{\rho }^{1.63}$ and ${\rho }^{1.66}$, respectively. The scaling exponent *b* is very close to the value obtained in experiment^32^ which is approximately 1.6^32^. The agreement between the present results and experiment for the value of *b* demonstrates that the fractal dimension of the aerogel reproduced in the present simulation is comparable to the practical one, which guarantees the reliability of the simulation results. The coefficient $\alpha$ in Eq. (17) is related to the structure parameters of the aerogel such as pore size. The pore size distribution (PSD) in the present simulation was analyzed by PSDsolv developed by Bhattacharya^49^, and an example is shown in Fig. 5b. As can be seen, the maximum pore diameter appearing in the most expanded system is about 17 nm, which is 10 times bigger than that reproduced in a previous simulation study^34^. However, the pore size generated in the present study is still much smaller than the pore size of aerogels measured in the experiment with the same density (e.g., $D$ = 81.9 nm at $\rho =$ 0.0557 ${\text{g}}\,{\text{cm}}^{ - 3}$ ^20^). Therefore, the absolute value of ${\lambda }_{s}$ from the MD simulation overestimates the experimental data^20^ by almost 4 times, as shown in Fig. 5a. To generate a porous structure with a larger pore size, aiming at improving the consistency of the prediction by the MD simulation and the experimental measured thermal conductivity, a bigger simulation box and a larger number of atoms are required, which cost unaffordable computing power. One possible way is to develop truncated or coarse-grained potential. Wolf shifted BKS potential used by Gonçalves et al.^50^ provides an alternative but its reliability in thermal characterization still needs to be validated since the performance of BKS potential is not good. Nevertheless, the results of the present study are as accurate as can be achieved by MD simulation and are beneficial for research of aerogels at the tens of nanometer scale.

As shown in the inset of Fig. 5a, ${\lambda }_{s}$ tends to be lower at higher defect concentration when the densities are below 0.2 ${\text{g}}\,{\text{cm}}^{ - 3}$. It can be deduced that the defect scattering of oscillators cannot be ignored when the aerogel is highly porous or when the skeleton has a smaller size. Considering the shorter mean free path in thinner skeletons, an increase in multiple scattering events at the solid–gas interface considerably attenuates the energy transferred by oscillators. This results in lower heat conduction through the solid skeleton and, thereby, lower conductive thermal conductivity. On the contrary, when the density is above 1 g cm^−3^, ${\lambda }_{s}$ is higher for solid with higher defect concentration. The reason is that the influence of defect scattering on the thermal conductivity is not dominant and the pore size is playing a leading role at high density. Note that, for the same skeletons (the volumes of simulation boxes are the same), the defective structure has lower density since it is generated through deleting atoms from the perfect structure. In other words, at a given density, the structure with higher defect concentration is less expanded and has smaller pore size. Therefore, higher ${\lambda }_{s}$ for a more defective system is to be expected.

### Gas conductivity ${\lambda }_{g}$

The gas conductivity ${\lambda }_{g}$ for different gas species filling the silica skeletons was calculated. To eliminate the solid gas coupled effect and study the separate gas conductivity, silica (silicon and oxygen atoms) in the porous structure were fixed in the simulation, with the vibration of each atom in the solid phase being zero. In this case, thermal energy is transferred solely through the gas molecules, i.e., the kinetic energy exchange is applied only on gas molecules, and the simulated conductive thermal conductivity is the gas thermal conductivity. It should be noted that ${\lambda }_{g}$ in the present study is actually the separated contribution of gas conduction to the effective conductivity. For aerogels with compromising inter-connected pores, MD simulation can provide direct calculation of the effective thermal conductivity, while the Kaganer^14^ and Zeng^51^ models are weak in doing this since they are designed to evaluate the conductivity of gas in a single confined space.

**Figure 6:**
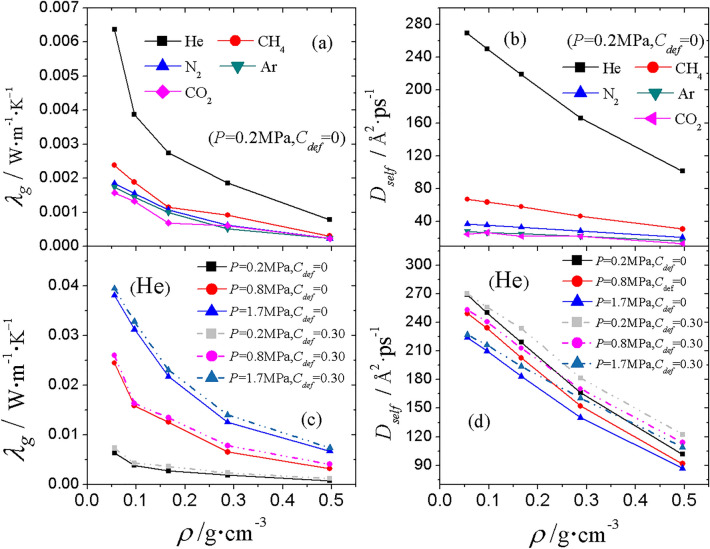
The gas conductivity ${\lambda }_{g}$ for (**a**) different gas species and under (**c**) different pressures and defect concentrations $C_{def}$. Self-diffusion coefficient ${D}_{self}$ of gas molecules for (**b**) different gas species and under (**d**) different pressures and defect concentrations $C_{def}$. For clarity, the results for perfect ($C_{def}$ = 0) and defective structures were aligned to the same $\rho$ which is the density of the original skeleton before deleting atoms.

The results are shown in Fig. 6. It can be seen from Fig. 6a that ${\lambda }_{g}$ decreases with increasing aerogel density. The reason is that the Knudsen effect is enhanced as the pore size becomes smaller. In the same skeleton, ${\lambda }_{g}$ for He is the largest, followed by CH_4_, N_2_, Ar, with ${\lambda }_{g}$ for CO_2_ the smallest. Since the heat conduction is energy transport by atoms, ${\lambda }_{g}$ is related to the mobility of gas molecules. The higher mobility means that the energy carried by gas molecules can be transported further per unit time, which promotes thermal conduction.

The self-diffusion coefficients ${D}_{self}$ were used to describe the mobility of gas molecules. ${D}_{self}$ was calculated by using Einstein equations^52–54^:

18 $$D_{self} = \mathop {\lim }\limits_{t \to \infty } \frac{{\left\langle {\left[ {r_{i} (t) - r_{i} (0)} \right]^{2} } \right\rangle }}{2dt},$$

19 $$D_{self} = \mathop {\lim }\limits_{t \to \infty } \frac{{\left\langle {(x_{i} (t) - x_{i} (0))^{2} + (y_{i} (t) - y_{i} (0))^{2} + (z_{i} (t) - z_{i} (0))^{2} } \right\rangle }}{6t},$$

where $r_{i} (t)$ and $r_{i} (0)$ are the positions of atom* i* at time *t* and 0, respectively, $\left[ {r_{i} (t) - r_{i} (0)} \right]^{2}$ is the mean-square displacement (MSD), x, y and z are the lateral Cartesian coordinates for the center of mass of gas molecules, $d$ equals 3 for the situation where three dimensions of motion are concerned, and $\left\langle \ldots \right\rangle$ denotes an ensemble average. In Fig. 6b, the self-diffusion coefficients ${D}_{self}$ of gas molecules are displayed. At a given pressure, the diffusion coefficient ${D}_{self}$ of each gas species decreases almost linearly as the aerogel density increases. The movement of gas molecules are constrained by the narrow gap between solid skeletons, hence the conduction by gas is retarded. Among all the gas species, He has the largest mobility because of its smaller molecular weight. As expected, CO_2_ has the largest molecular weight, and hence the lowest mobility and thermal conductivity.

The influence of the defect concentration $C_{def}$ on the gas conductivity ${\lambda }_{g}$ was also analyzed. ${\lambda }_{g}$ in the systems with different $C_{def}$ and under varied pressure was calculated. The gas conductivity of He is the highest among the 5 gas species, so ${\lambda }_{g}$ for He was chosen as an example for its lower relative error. The results are shown in Fig. 6c. When the defect of 0.3 is introduced, ${\lambda }_{g}$ increases slightly. This enhancement is also from higher gas mobility. The results of self-diffusion coefficient ${D}_{self}$ illustrated in Fig. 6d show that ${D}_{self}$ of He is slightly larger in highly defective structures ($C_{def}$ = 0.3) than that in a perfect structure ($C_{def}$ = 0) at a given expanded volume. The reason is that the inclusion of defects enlarges the size of micro pores. Therefore, the difference between ${D}_{self}$ with and without defects is distinct when the aerogel is dense and most of the pores are on the sub-nano scale. As the aerogel density reduces, where the pore size is in the nanometer range, the improvement of ${D}_{self}$ with higher defect concentration becomes less obvious.

In addition, the capability for heat conduction is not only associated with the molecule mobility, but also proportional to the number of gas molecules in unit length. As demonstrated in Fig. 6d, with the pressure increasing from 0.2 to 1.7 MPa, ${\lambda }_{g}$ systematically increases by about 10 times, but the mobility of gas indicated by ${D}_{self}$ of He becomes lower under increased pressure. The increased thermal conductivity under higher pressure is not primarily caused by higher mobility, but it is dominated by the effect of higher number density of gas molecules. Under high pressure, the density of gas rises substantially but the higher number density of gaseous molecules increases the possibility of gas–gas intermolecular collisions. Consequently, the mobility is restricted and the gas conductivity ${\lambda }_{g}$ increases. This tendency is the same for the other 4 species of gas (not shown).

**Figure 7:**
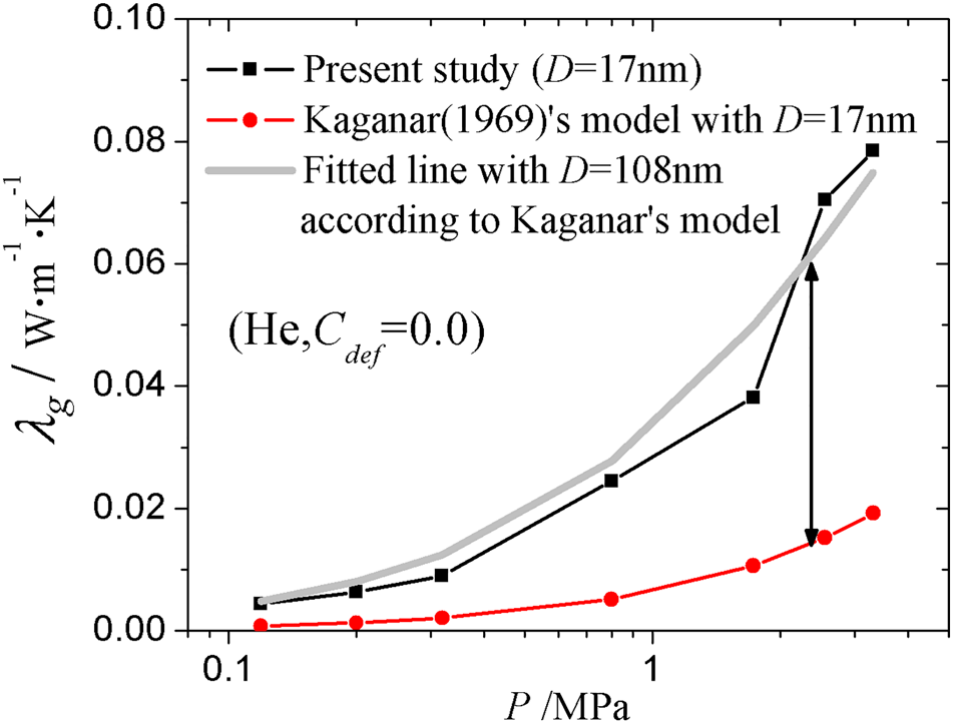
The measured ${\lambda }_{g}$ of aerogels with pore size of *D* = 17 nm from the molecular dynamics simulation, as well as the predicted ${\lambda }_{g}$ based on Kaganar’s model with pore size *D* = 17 nm and the fitted characteristic size *D* = 108 nm as a function of the gas pressure.

The measured ${\lambda }_{g}$ in the present MD simulation study and the predicted ${\lambda }_{g}$ based on the Knudsen formula, i.e., Kaganar’s model Eq. (2) under different pressure were compared, as shown in Fig. 7. The case for He with aerogel density $\rho$ = 0.0557 g cm^3^ and $C_{def}$ = 0.0 was taken as an example. When the pore size is set as *D* = 17 nm, which is the diameter of the biggest pore obtained by PSDsolv (see Fig. 5b), the predicted ${\lambda }_{g}$ based on Eq. (2) is much lower than the simulated ${\lambda }_{g}$ in the present work. To fit the simulated results into the Knudsen formula, the pore size *D* has to be approximately 108 nm, which is 5–6 times larger than the actual *D*. This suggests that the traditional Knudsen formula is no longer applicable to describe the gas thermal conductivity in an open cell porous structure. The use of the Knudsen formula may lead to underestimation of ${\lambda }_{g}$ without an appropriate choice of *D*. The characteristic size *D* that has the Knudsen effect on gas should be larger than the pore size because the pores in aerogel are inter-connected and gas molecules can move beyond the pore spaces.

### Effective thermal conductivity of solid ${\lambda }_{s}^{e}$

To measure the coupled solid–gas conduction effect, gas molecules and silica atoms were all released and moved under the interactions between them in the MD simulation. Therefore, energy transport occurs in and between gas and solid phases. The kinetic energy exchange was applied on the solid first to compute the effective solid conductivity ${\lambda }_{s}^{e}$ under the influence of gas. Under higher pressure, the larger number density of gas molecules increases the solid–gas interfacial collisions at the interface. More energy can be conducted through the porous structure by solid–solid interatomic interactions in the solid, gas–gas intermolecular interactions in the gas, and solid–gas ballistic interfacial interactions at the porous surface. Therefore, the coupled solid–gas conduction effect can be determined by analyzing the difference between the solid conductivity and the effective solid conductivity ${\lambda }_{s}^{e}-{\lambda }_{s}$.

**Figure 8:**
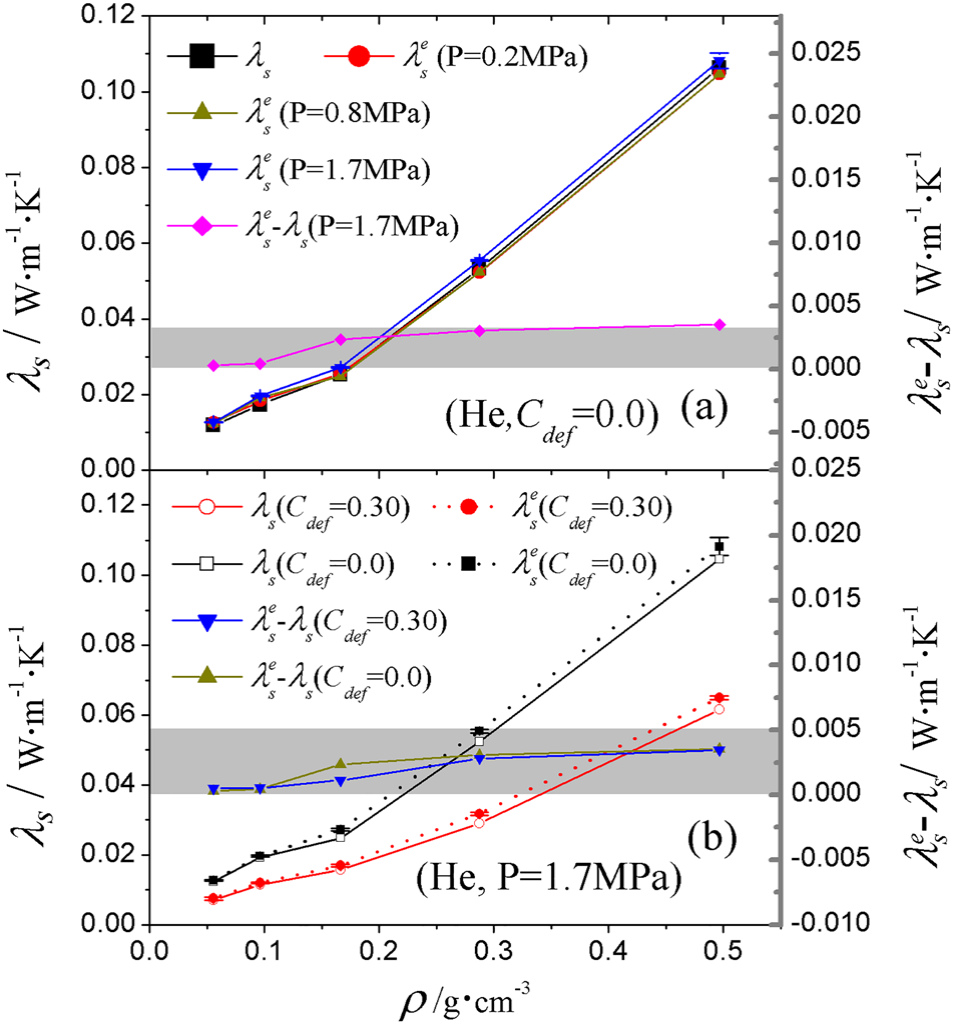
Effective solid thermal conductivity ${\lambda }_{s}^{e}$ in He atmosphere as a function of (**a**) gas pressure and (**b**) defect concentration $C_{def}$. For clarity, the results for perfect ($C_{def}$ = 0) and defective structures were aligned to the same $\rho$.

For aerogels consisting of skeletons without defects ($C_{def}$ = 0), as shown in Fig. 8a, the effective thermal conductivity of the solid ${\lambda }_{s}^{e}$ is not sensitive to the species of gas. While the gas conductivity ${\lambda }_{g}$ varies with gas species, ${\lambda }_{s}^{e}$ for the same skeleton fluctuates in a very narrow range (shown by the error bars). It can be speculated that the solid thermal conduction is influenced by the solid–gas interaction but is almost independent of the gas–gas interaction that determines the gas thermal conductivity. Also, the solid–gas coupling effect is negligible, as reflected by the difference between ${\lambda }_{s}^{e}$ and ${\lambda }_{s}$. For example, the relative difference between ${\lambda }_{s}^{e}$ under gas pressure of 0.2 MPa and ${\lambda }_{s}$ without gas is within ± 1.8% over the whole density range studied. Such a small discrepancy could be attributed to statistical error. This suggests that the solid thermal conduction can hardly be affected by rarefied gas. Since heat is conducted through atom vibration in a solid, the number density of molecules in rarefied gas is so small that collisions between gas and silica are less frequent and have little influence on the atom vibration in the solid. Some theories^21,55,56^ claim that the gas molecules near the contact point of solid particles (i.e. the skeleton necks) are in a quasi-lattice vibrating state, and these solid-like gas molecules contribute to the solid–gas coupled thermal conductivity, effectively through bridging the adjacent solid particles. However, the result here indicates that this bridging effect is not significant, at least on a small scale. With the pressure increasing from 0.2 to 1.7 MPa, the enhancement of solid conductivity due to the coupling effect is still weak. The maximum relative difference between ${\lambda }_{s}^{e}$ and ${\lambda }_{s}$ is less than 7%. However, a tendency can be seen for ${\lambda }_{s}^{e}-{\lambda }_{s}$ to grow with increasing gas pressure.

Considering the solid conductivity of aerogels at a fixed gas pressure, Fig. 8b shows the results when defects are introduced into the solid. When the defect concentration is $C_{def}$ = 0.30, ${\lambda }_{s}^{e}-{\lambda }_{s}$ does not change much compared with the value for the same skeleton without defects, even the solid conductivity is lower when the defect level is high. In other words, reducing the solid conductivity is not a decisive way to suppress the solid–gas coupling effect.

### Effective gas conductivity ${\lambda }_{g}^{e}$

**Figure 9:**
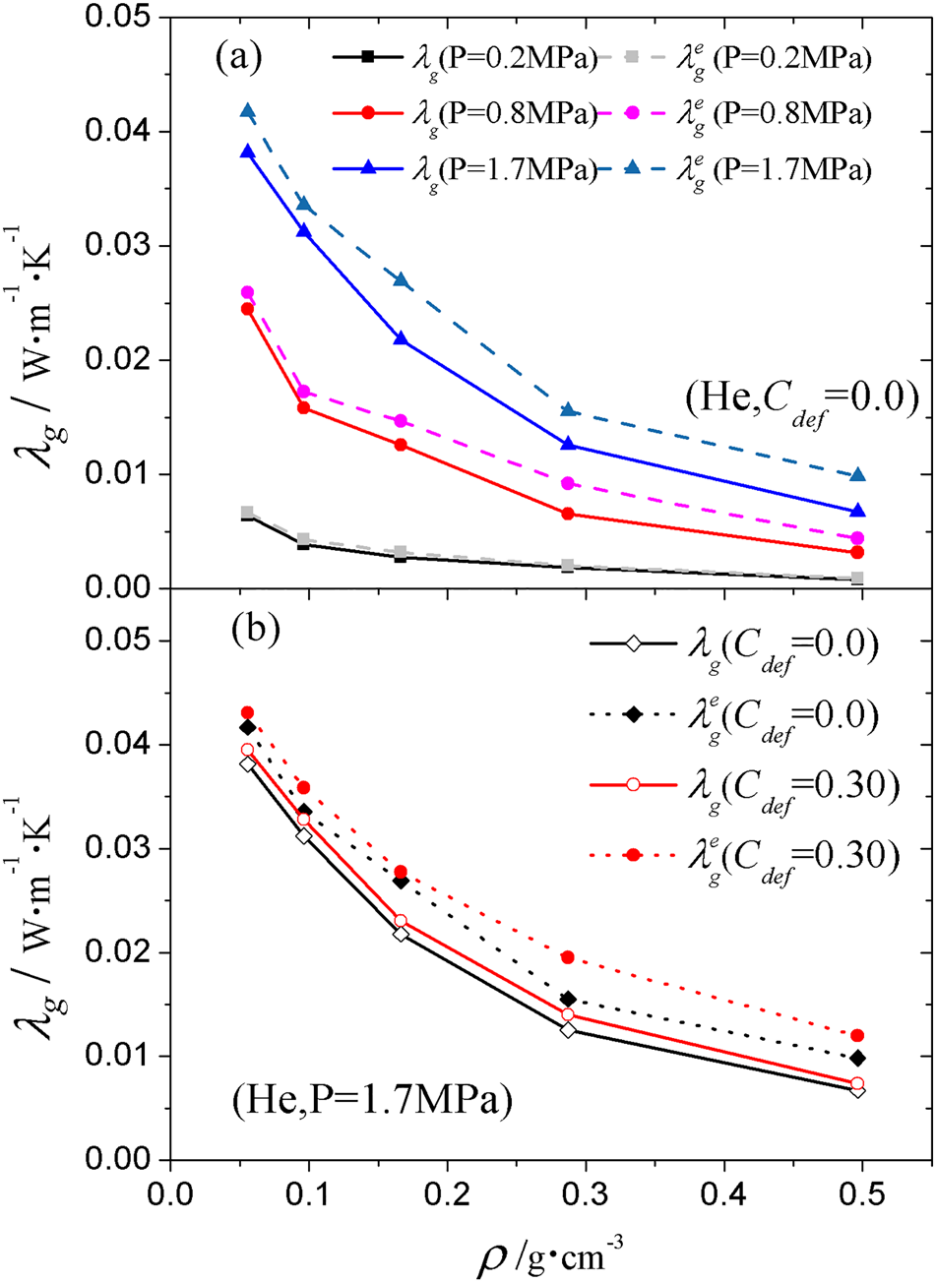
Effective gas thermal conductivity ${\lambda }_{g}^{e}$ of He as a function of (**a**) gas pressure and (**b**) defect concentration $C_{def}$. For clarity, the results for perfect ($C_{def}$ = 0) and defective structures were aligned to the same $\rho$.

The effective gas conductivity ${\lambda }_{g}^{e}$ under the influence of the solid was computed through applying kinetic energy exchange on the gas. Such influence can be determined by the difference between the gas conductivity and the effective gas conductivity ${\lambda }_{g}^{e}-{\lambda }_{g}$. Figure 9 shows the results of ${\lambda }_{g}^{e}$ and ${\lambda }_{g}$ for helium under different pressures. As shown in Fig. 9a, ${\lambda }_{g}^{e}$ is systematically higher than ${\lambda }_{g}$ in skeletons with different densities. This suggests that the solid–gas coupling, not only affects the effective solid conductivity, but also the effective gas conductivity. If the silica is fixed where ${\lambda }_{s}=0$, some gas molecules are blocked to transport energy by fixed solid in their motion path. After the silica is released, this part of the energy can be transported through collisions between gas and solid molecules.

Under the same pressure, ${\lambda }_{g}^{e}-{\lambda }_{g}$ for different aerogel densities is very close, even although ${\lambda }_{g}$ varies dramatically with the aerogel’s bulk density. The reason is that thermal transport between solid and gas is through ballistic collisions at the interface. At a higher aerogel bulk density and a smaller pore size, gas–gas intermolecular interactions become lower because of the lower volume fraction of gas. However, solid–gas ballistic collisions between solid and gas are higher because of the larger surface area in the smaller sized porous structure. On the other hand, the opposite is true for lower aerogel bulk density. Thus, there is no explicit correlation between ${\lambda }_{g}^{e}-{\lambda }_{g}$ and ${\lambda }_{g}$.

Under higher pressure, with the number density of gas molecules increasing, the coupling conductivity ${\lambda }_{g}^{e}-{\lambda }_{g}$ is expected to increase due to higher frequency of collisions. This expectation is confirmed by the results shown in Fig. 8a. Under 0.2 MPa, 0.8 MPa and 1.7 MPa, ${\lambda }_{g}^{e}-{\lambda }_{g}$ for He is about ${0}{\text{.0003}}\,{\text{W}}\,{\text{m}}^{{{ - }1}} \,{\text{K}}^{{{ - }1}}$, ${0}{\text{.002}}\,{\text{W}}\,{\text{m}}^{{{ - }1}} \,{\text{K}}^{{{ - }1}}$ and ${0}{\text{.004}}\,{\text{W}}\,{\text{m}}^{{{ - }1}} \,{\text{K}}^{{{ - }1}}$, respectively. In addition, the value of ${\lambda }_{g}^{e}-{\lambda }_{g}$ for CH_4_, N_2_, Ar and CO_2_ is a little lower than for He (not shown), suggesting that ${\lambda }_{g}^{e}-{\lambda }_{g}$ is sensitive to the gas species. Similar to the situation for solid conductivity, the inclusion of defects does not cause much variation in ${\lambda }_{g}^{e}-{\lambda }_{g}$. As shown in Fig. 9b, ${\lambda }_{g}^{e}-{\lambda }_{g}$ for the system with $C_{def}$ = 0.30 is of the same order of magnitude as that for $C_{def}$ = 0.00. Overall, the most effective factor to influence ${\lambda }_{g}^{e}-{\lambda }_{g}$ is gas pressure.

## Conclusions

Molecular dynamics simulations on porous silica aerogels were conducted to investigate their thermal conduction properties. The fractal aerogel structures were generated through negative pressure rupture (NPR). The pore size reproduced was 10 times larger than in previous studies, making it possible to include the gas conductivity in the investigation of aerogel thermal conductivity. The separated and coupled solid–gas thermal conductivities were obtained using the M–P method. The separated solid conductivity of the simulated skeletons ${\lambda }_{s}$ was measured under no gas pressure. The results show that ${\lambda }_{s}$ decreases with increase of density $\rho$. There is an exponential relationship between ${\lambda }_{s}$ and $\rho$ where the exponent b is approximately 1.6. In the density range studied, *b* increases with increase of defect concentration *C*. The separated gas conductivity ${\lambda }_{g}$ was measured with the silica atoms fixed (${\lambda }_{s}=0$). The results suggest that ${\lambda }_{g}$ is related to the diffusion coefficient of gas molecules, therefore, ${\lambda }_{g}$ increases in the presence of low molecular weight gas, large pore size, low aerogel bulk density or high defect concentration in the solid. Also, ${\lambda }_{g}$ rises as the gas pressure increases because the larger number density of gas molecules under higher pressure means greater capacity to transport energy. The use of Kaganer’s model with the pore size *D* of the open cell structure itself may lead to underestimation of ${\lambda }_{g}$. The characteristic size *D* that has the Knudsen effect on gas should be much larger than the pore size because the gas molecules can move beyond the pore space.

In the last step, the silica and gas were all released to move under the interactions between them so that the solid–gas coupling effect was included. The effective solid conductivity ${\lambda }_{s}^{e}$ was calculated through applying kinetic energy exchange on the solid only and recording the temperature gradient. The results show that the enhancement of solid conductivity due to the coupling effect is negligible in a rare field gas. Therefore, contrary to some previous studies, the gas molecules located in the narrow corner created by adjacent solid particles could not contribute much to the solid–gas coupling thermal conductivity. Increased gas pressure leads to a slightly stronger solid–gas coupling effect, while the defect concentration has little influence on coupling. The effective gas conductivity ${\lambda }_{g}^{e}$ was calculated in a similar way. It was found that the coupling effect ${\lambda }_{g}^{e}-{\lambda }_{g}$ for different aerogel densities changes very little at the same pressure. The reason is that ${\lambda }_{g}$ and ${\lambda }_{s}$ are affecting ${\lambda }_{g}^{e}-{\lambda }_{g}$ simultaneously, resulting in a similar effect between low density where ${\lambda }_{g}$ is higher and ${\lambda }_{s}$ is lower, and high density where ${\lambda }_{g}$ is lower and ${\lambda }_{s}$ is higher. Increasing the gas pressure results in higher number density of gas, and thus higher ${\lambda }_{g}^{e}-{\lambda }_{g}$. Overall, the most significant factor that influences ${\lambda }_{g}^{e}-{\lambda }_{g}$ is gas pressure. As a conclusion, when decreasing the gas pressure, the number density of gaseous molecules becomes smaller, and both gas–gas intermolecular interaction and solid–gas ballistic interaction at the interface are reduced. Therefore, decreasing the gas pressure is an efficient way to reduce the solid–gas-coupling conductivity, from the perspective of both effective solid conductivity and effective gas conductivity. These findings could be used in multi-scale simulations and would be beneficial for improving the accuracy of predictions of aerogel thermal conductivity.
